# Association of physical activity with vascular aging in a population with intermediate cardiovascular risk, analysis by sex: MARK study

**DOI:** 10.1186/s13293-022-00456-w

**Published:** 2022-08-20

**Authors:** Leticia Gómez-Sánchez, Emiliano Rodríguez-Sánchez, Rafel Ramos, Ruth Marti, Marta Gómez-Sánchez, Cristina Lugones-Sánchez, Olaya Tamayo-Morales, Susana Gonzalez Sánchez, Fernando Rigo, Luis García-Ortiz, Manuel A. Gómez-Marcos, the MARK Investigators, Rafel Ramos, Rafel Ramos, Ruth Martí, Dídac Parramon, Anna Ponjoan, Miquel Quesada, Maria Garcia-Gil, Martina Sidera, Lourdes Camós, Fernando Montesinos, Ignacio Montoya, Carlos López, Anna Agell, Núria Pagès, Irina Gil, Anna Maria-Castro, Fernando Rigo, Guillermo Frontera, Antònia Rotger, Natalia Feuerbach, Susana Pons, Natividad Garcia, John Guillaumet, Micaela Llull, Mercedes Gutierrez, Cristina Agudo-Conde, Leticia Gómez-Sanchez, Carmen Castaño-Sanchez, Carmela Rodriguez-Martín, Benigna Sanchez-Salgado, Angela de Cabo-Laso, Marta Gómez-Sánchez, Emiliano Rodriguez-Sanchez, Jose Angel MaderueloFernandez, Emilio Ramos-Delgado, Carmen Patino-Alonso, Jose I. Recio-Rod-riguez, Manuel A. Gomez-Marcos, Luis Garcia-Ortiz

**Affiliations:** 1 Primary Care Research Unit of Salamanca (APISAL), Biomedical Research Institute of Salamanca (IBSAL), Avenida de Portugal 83, 37005 Salamanca, Spain; 2 Health Service of Castilla and Leon (SACyL), Avenida de Portugal 83, 37005 Salamanca, Spain; 3 Department of Medicine, University of Salamanca, Alfonso X el Sabio s/n, 37007 Salamanca, Spain; 4 Red de Investigación en Cronicidad, Atención Primaria y Promoción de la Salud (RICAPPS) (RD21/0016), Barcelona, Spain; 5 Unitat of Suport the Recerca of Girona, Institut Universitari d’Investigacio in Atencion Primària Jordi Gol (IDIAP Jordi Gol), Girona, Spain; 6 Institut d’InvestigacioÂ Biomèdica of Girona Dr. Josep Trueta (IDBGI), Girona, Spain; 7 Departament of CiènciesMèdiques, Facultat of Medicina, Universitat of Girona, Girona, Spain; 8 San Agustin Health Center, Illes Balears Health Service (IBSALUT), Palma of Mallorca, Spain; 9 Department of Biomedical and Diagnostic Sciences, University of Salamanca, calle Alfonso X el Sabio s/n, 37007 Salamanca, Spain

**Keywords:** Vascular aging, Physical activity, Spanish population

## Abstract

**Background:**

The aim of this study was to analyze the association of physical activity and its intensity with arterial stiffness and vascular aging and differences by sex in a Spanish population with intermediate cardiovascular risk.

**Methods:**

Cross-sectional study. A total of 2475 individuals aged 35–75 years participated in the study. Brachial–ankle pulse wave velocity (baPWV) was measured using a VaSera VS-1500^®^ device. Based on the age and sex percentile presented by the participants, the latter were classified as follows: those with a percentile above 90 and presenting established cardiovascular disease were classified as early vascular aging (EVA); those with a percentile between 10 and 90 were classified as normal vascular aging (NVA) and those with a percentile below 10 were classified as healthy vascular aging (HVA). Physical activity was analyzed through the short version of the Minnesota Leisure Time Physical Activity Questionnaire (MLTPAQ).

**Results:**

The mean age of the participants was 61.34 ± 7.70 years, with 61.60% men. Of the total sample, 86% were sedentary (83% men vs 90% women). The total physical activity showed a negative association with baPWV (*β* = − 0.045; 95% CI − 0.080 to − 0.009). Intense physical activity showed a negative relationship with baPWV (*β* = − 0.084; 95% CI − 0.136 to − 0.032). The OR of the total physical activity and the intense physical activity carried out by the subjects classified as NVA with respect to those classified as HVA was OR = 0.946; (95% CI 0.898 to 0.997) and OR = 0.903; (95% CI 0.840 to 0.971), and of those classified as EVA it was OR = 0.916; (95% CI 0.852 to 0.986) and OR = 0.905; (95% CI 0.818 to 1.000). No association was found with moderate- or low-intensity physical activity.

**Conclusions:**

The results of this study suggest that, when intense physical activity is performed, the probability of presenting vascular aging is lower. In the analysis by sex, this association is only observed in men.

**Supplementary Information:**

The online version contains supplementary material available at 10.1186/s13293-022-00456-w.

## Highlights

- This study is the first to find a negative association between total physical activity and arterial stiffness and vascular aging in adults with intermediate cardiovascular risk.
- This study also shows that the association of physical activity with arterial stiffness and vascular aging in adults with intermediate cardiovascular risk varies depending on its intensity, with a negative association being found only with intense physical activity.
- In the analysis by sex, the results found suggest that the association is greater in men.

**Supplementary Information:**

The online version contains supplementary material available at 10.1186/s13293-022-00456-w.

## Background

Physical activity performed regularly at moderate–high intensity reduces the early morbidity and mortality of a large number of cardiometabolic diseases, tumors and mental diseases. Similarly, in both primary and secondary prevention, physical activity reduces or improves the prognosis of numerous medical affectations, increasing the quality of life of the people who practice it, with the corresponding benefits for the healthcare system and society [1–7]. Thus, most international guides report about the type, amount and intensity of the physical activity necessary to maintain and improve population health [2, 8], recommending 150 min/week of intense-moderate physical activity and considering this the minimum required to obtain health benefits. Moreover, recent evidence suggests that including low-intensity activities, at least for currently inactive populations, can be beneficial for health [3, 8].

Arterial stiffness, evaluated through brachial–ankle pulse wave velocity (baPWV), increases with age and predicts morbimortality by cardiovascular diseases [9]. Several studies and systematic reviews conducted in different populations indicate that physically active individuals have a lower arterial stiffness than sedentary individuals, suggesting that regular physical activity is an effective index of arterial stiffness [10–12]. However, depending on its intensity and type, the effect of physical activity on arterial stiffness is still controversial. Thus, some authors propose that the benefit is only obtained with exercises of moderate–high intensity, and that such benefit can vary depending on the type of exercise performed [13–15] and by sex [16]. Arterial stiffness is associated with the appearance of vascular aging [17, 18], reflecting the dissociation between chronological age and biological age of the main arteries, with their alteration preceding the appearance of cardiovascular events [17–19]. In recent decades, epidemiological studies have been carried out to determine the key factors of vascular aging, which has gained great interest, as it shows a stronger relationship with the morbimortality by cardiovascular diseases than biological aging [17, 18]. These studies suggest that vascular aging is healthier in the Spanish population than in other countries. Thus, in a general Spanish population without prior cardiovascular disease, a study showed that vascular age and heart age were − 3.08 ± 10.15 and − 2.98 ± 10.13 years younger, respectively. Likewise, 63.2% (men: 59.3%; women 67.1%) and 66.11% (men: 52.0%; women 71.1%) had vascular and heart ages below their chronological age [20]. These results are more favorable than those published in the review by Groenewegen et al. [21], which found that the difference between vascular age or heart age and chronological age was between 2 and 26 years higher, results consistent with those published in various studies [22–25]. Along the same lines, a second study carried out on a Spanish population analyzed vascular aging through arterial stiffness measured with the cfPWV and found that one in ten had HVA and one in five EVA. The prevalence of EVA was higher in men, and vascular aging deteriorated with increasing sedentary time and decreasing physical activity, as measured objectively over a week with an accelerometer [26]. These data are generally more favorable than those published in other countries [27–31]. However, it should be noted that the prevalences found in previous studies are not comparable, given that the criteria used to define HVA and EVA, the distribution of the population by age and sex, and their characteristics regarding the presence of risk factors and morbidity vary across the studies analyzed.

However, depending on the intensity and type of physical activity, and sex, its effect on arterial stiffness may vary. Thus, Vandercappellen et al. [13] examined the association of the amount and pattern of intense physical activity with arterial stiffness in the Maastricht study. They reported that while a negative association between intense physical activity and arterial stiffness was found, this was not the case with regular or weekend physical activity. Königstein et al. [14] evaluated the effects of light, moderate and vigorous physical activity independently of cardiorespiratory fitness on arterial stiffness, finding a reduction of 0.32 m/s for every 10-min increment in moderate or vigorous intensity physical activity per day, while physical activity of light intensity had no relevant effect on baPWV. Finally, in a review carried out by Figueroa et al. [15], aiming to examine the effects of resistance training intensity on arterial stiffness and blood pressure (peripheral and central) in young and older adults, it was found that high-intensity resistance training could decrease systemic arterial stiffness in healthy young adults, or not affect arterial stiffness in middle-aged and older adults. On the other hand, several authors have described differences between the sexes. Thus, Seals et al. [32] found that regular aerobic exercise improved endothelial function and vascular aging in men (by reducing oxidative stress and preserving NO bioavailability), but not consistently in estrogen-deficient post-menopausal women [32]. Conversely, Collier et al. [16] found that moderate-intensity physical exercise may be more favorable for women, as a treatment option for hypertension, due to greater reductions in diastolic blood pressure and increase in flow-mediated arterial dilation without increase in arterial stiffness compared to men. For all the above, the aim of this study was to analyze the association of physical activity and its intensity with arterial stiffness and vascular aging in a Spanish population with intermediate cardiovascular risk and explore the existence of differences between sexes.

## Methods

### Study design and population

The “improving intermediate risk management” study (MARK study) is a multicentre cross-sectional study registered in ClinicalTrials.gov: NCT01428934 [33].

The participants were recruited by family doctors and selected by random sampling among individuals visiting primary healthcare centers of three autonomous communities of Spain between July 2011 and June 2013. Inclusion criteria: (1) age between 35 and 75 years; (2) intermediate cardiovascular risk defined with equation A (5–15% coronary risk estimated with Framingham’s risk equation adapted to the Spanish population [34]), B (1–5% vascular mortality risk in 10 years estimated with the equation for the European population [35]), or C (moderate risk following the criteria of the European Society of Hypertension for the management of arterial hypertension [36]); and (3) signed the informed consent before being included in the study. Exclusion criteria: terminal disease, hospitalization, or history of atherosclerosis. A total of 2495 individuals were recruited. This manuscript presents an analysis of 2475 individuals recruited in the MARK study, excluding 20 individuals for not having their baPWV measured.

To estimate the power of the sample, we considered the difference in the main variable, total PA, between the HVA group (241 subjects) and the NVA group (1927 subjects) and between the HVA group (241 subjects) and the EVA group (307 subjects). Therefore, for a bilateral contrast and assuming an alpha risk of 0.05, in the first case, with a SD of 2525, the estimated power was 32% and in the second case, with a SD of 2522, the estimated power was 58%.

A detailed description of the procedures followed by the gathering of clinical data, anthropometric measures, cardiovascular risk factors and laboratory tests has previously been published in the protocol of the study [33].

### Ethical approval and consent to participate

Before being included in the study, the participants were briefed on all the procedures that they would be subjected to. This study was revised and approved by the Research Ethics Committee of the Jordi Gol Primary Healthcare Research Institute, the Drug Research Ethics Committee of Salamanca, and the Research Ethics Committee of Palma de Mallorca. During the realization of the study, the rules of the Declaration of Helsinki were followed [37].

### Variables and measurement instruments

Before starting the study, healthcare professionals were trained in each data-gathering center to record the measurements and complete the questionnaires following a standardized protocol.

### Evaluation of physical activity

The physical activity performed was recorded using the short version of the Minnesota Leisure Time Physical Activity Questionnaire (MLTPAQ) [38], which was designed to evaluate the amount and intensity of physical activity performed in leisure time (leisure and housekeeping activities), in the year prior to questionnaire completion, in middle-aged American men [39]. Elosua et al. validated the version of this questionnaire in Spanish men and women aged 18–61 years [40, 41]. This original questionnaire consists of 67 items, whereas the short version of the questionnaire used in this work [38] is made up of six items, which gather information about different types of physical activity: strolling, walking, farming/gardening, trekking, climbing stairs and practicing sports/exercising. The questionnaire evaluates the intensity of physical activity as low (strolling), moderate (walking, farming/gardening) and intense (trekking, climbing stairs and practicing sports/exercising).

The physical activity questionnaire was completed through a personal interview with qualified interviewers, who collected detailed information about the physical activity of the participants in the year before the study, the number of times they performed physical activity, and the average duration of each physical activity in each period. From the amount, type, frequency and duration of each session of physical activity, the baseline metabolic equivalents of task (MET) were calculated (MET) using the proposition of Ainsworth et al. [42] as a reference. One MET equals 1 kcal/kg of body weight per hour, and a consumption of 3.5 ml of oxygen/kg of body weight per min. The consumption of MET-min/14 days was estimated by multiplying the METs of each physical activity by its duration (in min), its accumulated frequency in the month prior to the interview (or habitual month), and by the months per year that the activity was performed, dividing by 365 days/year and multiplying by 14 days.

Following the recommendations of the American Heart Association, the participants were classified as sedentary if the moderate physical activity performed was < 675 METs min/week or the intense physical activity was < 420 METs min/week [43].

### Brachial–ankle pulse wave velocity (baPWV)

The baPWV was measured using a VaSera VS-1500^®^ device (FukudaDenshi), which employs an oscillometric method. The participants were requested to refrain from smoking or consuming caffeine one hour before the test and to attend the latter with comfortable clothing, remaining at rest for at least 10 min before conducting the measurements. The cuffs were adapted to the circumference of the arms and legs of both limbs. The electrodes were connected to the right and left arms and ankles of the participants, fixing a heart sound microphone to the chest, over the sternum, in the second intercostal space. The baPWV was calculated with the following equation: baPWV = (0.5934 × height (cm) + 14.4724)/tba (tba is the time interval between the arm and ankle waves) [44].

### Definition of vascular aging

In the selected sample, subjects with cardiovascular disease were identified. Therefore, in the first step, 20 individuals who presented cardiovascular disease (kidney disease, peripheral arteriopathy or heart failure), based on the criteria established in the 2013 clinical practice guide of the European Associations of Hypertension and Cardiology for the treatment of arterial hypertension [36], were classified as early vascular aging (EVA). Secondly, we classified the individuals using the 10th and 90th percentiles of baPWV by age group and sex. The individuals with baPWV values above the 90th percentile were considered to have EVA, those between the 10th and 90th percentiles were classified as having normal vascular aging (NVA), and those with values below the 10th percentile were considered to have HVA. The 10th and 90th percentile values of the ba-PWV by age and sex are shown in Additional file 2: Table S1.

### Statistical analysis

The continuous variables are shown as mean ± standard deviation, and the categorical variables are presented as number and percentage. The comparison of means between the two independent groups was carried out with Student’s *t*-test, and between more than two groups using a one-way analysis of variance. The post hoc analyses to explore the differences between more than two groups were conducted with the least significant difference test (LSD). In the comparison of categorical variables, the *χ*^2^ test and Fisher’s exact test were used. To analyze the association of physical activity with baPWV, two multiple linear regression analysis models were applied. Model 1, used baPWV as a dependent variable and total physical activity (measured in METs/min/week) as independent variable. Model 2 used baPWV as a dependent variable and intense physical activity, moderate physical activity and low physical activity (all included in the model and measured in METs/min/week) as independent variables. In both models, the adjustment variables we used were age (years), sex (woman = 1, man = 0), years of smoking, grams of alcohol per week, adherence to the Mediterranean diet, systolic blood pressure (mmHg), glycosylated hemoglobin (%), body mass index (kg/m^2^), antihypertensive drugs, and lipid-lowering and hypoglycemic drugs (Yes = 1 and Non = 0). To analyze the association of physical activity with vascular aging, we performed two multinomial logistic regression models (Model Fitting Criteria -2 Log Likelihood of Reduced Model). In Model 1, the degree of vascular aging was used as a dependent variable, coded as HVA = 1, NVA = 2 and EVA = 3, using HVA as the reference value and total physical activity (measured in METs/min/week) as independent variable. In Model 2, the degree of vascular aging was used as a dependent variable, coded as HVA = 1, NVA = 2 and EVA = 3, with HVA as the reference value and intense physical activity, moderate physical activity and light physical activity (all included in the model and measured in METs/min/week) as independent variables. Both models were controlled for age (years) and sex (woman = 1, man = 0), with years of smoking, grams of alcohol per week, adherence to the Mediterranean diet, type 2 diabetes mellitus, arterial hypertension, obesity, antihypertensive drugs, lipid-lowering drugs and hypoglycemic drugs (Yes = 1 and Non = 0) used as adjustment variables. The analyses were conducted globally and by sex. In the hypothesis test, an alpha risk of 0.05 was established as the limit of statistical significance. All the analyses were performed using the SPSS v.25 software for Windows (IBM Corp, Armonk, NY, USA).

## Results

### Clinical characteristics and vascular aging

The cardiovascular risk factors and diseases, lifestyles, total physical activity, intensity of the physical activity and vascular function of the participants are shown in Table 1 globally and by sex. The men performed more total physical activity, of high- and moderate-intensity, and consumed more alcohol and tobacco, whereas the women showed greater adherence to the Mediterranean diet (MD).

**Table 1:** General characteristics of the participants globally and by sex

	Global	(2475)	Men	(1524)	Women	(951)	*p* value
	Mean or *N*	SD or (%)	Mean or *N*	SD or (%)	Mean or *N*	SD or (%)	
Conventional risk factors							
Age, (years)	61.34	7.70	61.11	8.11	61.70	7.00	0.066
SBP, (mmHg)	137.25	17.37	139.09	17.05	134.32	17.48	< 0.001
DBP, (mmHg)	84.58	10.23	85.67	10.44	82.84	9.63	< 0.001
BP, (mmHg)	52.66	14.16	53.40	14.19	51.47	14.04	0.001
Hypertension, *n* (%)	1795	(72.5)	1172	(76.9)	623	(65.5)	< 0.001
Antihypertensive drugs, *n* (%)	1272	(51.4)	786	(50.4)	504	(53.0)	0.215
Total cholesterol, (mg/dl)	225.53	41.07	220.39	38.92	233.77	43.05	< 0.001
LDL cholesterol, (mg/dl)	139.88	34.97	138.34	34.26	142.32	35.96	0.006
HDL cholesterol, (mg/dl)	49.81	13.03	47.89	11.96	52.90	14.04	< 0.001
Triglycerides, (mg/dl)	146.21	96.48	150.99	105.90	138.59	78.63	0.001
Dyslipidemia, *n* (%)	1664	(67.2)	969	(63.6)	695	(73.2)	< 0.001
Lipid-lowering drugs, *n* (%)	717	(29.0)	419	(27.5)	298	(31.3)	0.045
FPG, (mg/dl)	107.98	34.83	107.76	33.90	108.35	36.29	0.683
HbA1c, (in %)	6.12	1.18	6.06	1.12	6.21	1.26	0.002
Diabetes mellitus, *n* (%)	842	(34.0)	493	(32.3)	349	(36.7)	0.029
Hypoglycaemic drugs, *n* (%)	511	(20.6)	289	(19.0)	222	(23.3)	0.009
Height, cm	164.56	9.27	169.69	6.78	156.34	6.36	< 0.001
Weight, kg	79.41	14.67	83.81	13.52	72.36	13.67	< 0.001
WC, (cm)	100.95	11.68	102.94	10.52	97.76	12.69	< 0.001
BMI, (kg/m^2^)	29.26	4.52	29.06	3.95	29.59	5.28	0.004
Obesity, *n* (%)	897	(26.2)	510	(33.5)	387	(40.7)	< 0.001
Abdominal obesity, *n* (%)	1546	(62.8)	797	(52.6)	749	(79.3)	< 0.001
Plasma creatine, (mg/dl)	0.85	0.23	0.94	0.24	0.71	0.13	< 0.001
GFR (ml/min/1.73 m^2^)	87.47	14.21	86.79	14.87	88.56	13.02	0.003
CVR, SCORE scale, (%)	3.55	2.73	4.48	2.93	2.05	1.40	< 0.001
Cardiovascular diseases							
Renal disease, *n* (%)	2	(0.1)	2	(0.1)	0	(0.0)	0.526
Peripheral arteriopathy, *n* (%)	13	(0.5)	11	(0.7)	2	(0.2)	0.150
Heart failure, *n* (%)	12	(0.5)	11	(0.7)	1	(0.1)	0.036
Lifestyles							
Years of smoker, (years)	31.66	12.82	31.95	12.78	30.74	12.92	0.113
Smoker, *n* (%)	710	(28.7)	486	(31.9)	224	(23.7)	< 0.001
Alcohol, (g/W)	71.87	116.74	102.01	132.71	23.58	58.86	< 0.001
Risk consumption (*n*, %)	334	(13.5)	284	(18.6)	50	(5.3)	< 0.001
MD, (total score)	5.18	1.73	5.09	1.78	5.32	1.63	0.002
Adherence MD, *n* (%)	1295	(52.4)	781	(51.2)	514	(54.0)	0.186
Total PA, (METs/m/W)	2462	2495	2864	2815	1817	1683	< 0.001
High-intensity PA, (METs/m/W)	917	1661	1096	1913	629	1087	< 0.001
Moderate-intensity PA, (METs/m/W)	811	1502	1012	1726	488	964	< 0.001
Low-intensity PA, (METs/m/W)	734	1003	755	1047	700	927	0.182
Sedentary, *n* (%)	2127	(85.9)	1269	(83.3)	858	(90.2)	< 0.001
Vascular function							
baPWV, (m/s)	14.87	2.63	14.82	2.65	14.93	2.60	0.313
baPWV yes sedentary, (m/s)	14.89	2.64	14.86	2.67	15.06	2.61	0.598
baPWV non sedentary MD, (m/s)	14.74	2.57	14.65	2.55	14.92	2.59	0.191

Values are means ± standard deviations for continuous data and number and proportions for categorical data

Risk alcohol consumption in women were ≥ 140 g/week and in men ≥ 210 g/week. Sedentary if the moderate physical activity performed is < 675 METs min/week or the intense physical activity < 420 METs min/week. Definition American Heart Association, 2007. Adherence MD ≥ 5

*N*, number; SD, standard deviation; g/W, grams/week; PA, physical activity; METs/m/W, basal metabolic rate/min/week; MD, Mediterranean diet; SBP, systolic blood pressure; DBP, diastolic blood pressure; BP, pulse pressure; LDL, low-density lipoprotein; HDL, high-density lipoprotein; FPG, fasting plasma glucose; HbA1c, glycosylated hemoglobin; WC, waist circumference; BMI, body mass index; CVR, cardiovascular risk; GFR, glomerular filtration; baPWV, brachial–ankle pulse wave velocity

*p* value: differences between men and women

Table 2 shows the total physical activity and its different levels of intensity as a function of the degree of vascular aging in the global sample and by sex. The individuals classified as HVA globally and in men performed more total physical activity and of high intensity compared to the individuals classified as NVA and EVA. No differences were found in the women.

**Table 2:** Characteristics of physical activity in participants with healthy, normal and early vascular aging globally and by sex

	HVA	NVA	EVA	*p* value
Global	(*n* = 241)	(*n* = 1927)	(*n* = 307)	
Total PA, (METs/m/W)^#^	2719 ± 2896	2462 ± 2479	2253 ± 2229	0.056
High-intensity PA, (METs/m/W)^*,#^	1214 ± 2234	885 ± 1600	886 ± 1485	0.014
Moderate-intensity PA, (METs/m/W)	844 ± 1606	827 ± 1518	678 ± 1296	0.256
Low-intensity PA, (METs/m/W)	660 ± 985	750 ± 551	688 ± 884	0.294

Men	(*n* = 148)	(*n* = 1166)	(*n* = 210)	
Total PA, (METs/m/W)^*,#^	3386 ± 3353	2868 ± 2815	2471 ± 2308	0.010
High-intensity PA, (METs/m/W)^*,#^	1577 ± 2640	1052 ± 1847	1005 ± 1603	0.005
Moderate-intensity PA, (METs/m/W)^¥^	1101 ± 1902	1044 ± 1758	772 ± 1367	0.090
Low-intensity PA, (METs/m/W)	708 ± 1059	772 ± 1063	693 ± 943	0.510

Women	(*q* = 93)	(*n* = 761)	(*n* = 97)	
Total PA, (METs/m/W)	1658 ± 1435	1841 ± 1671	1783 ± 1980	0.598
High-intensity PA, (METs/m/W)	738 ± 1157	628 ± 1071	629 ± 1158	0.997
Moderate-intensity PA, (METs/m/W)	436 ± 820	495 ± 962	475 ± 1106	0.090
Low-intensity PA, (METs/m/W)	584 ± 854	716 ± 957	687 ± 745	0.845

Values are represented as mean (standard deviation) for continuous data

Differences among groups: continuous variables analysis of variance and post hoc using the DMS tests

PA, physical activity; METs/m/W, basal metabolic rate/min/week; EVA, early vascular aging; HVA, healthy vascular aging; NVA, normal vascular aging

^*^p value < 0.05 between HVA and NVA

^¥^*p* value < 0.05 between NVA and EVA

^#^*p* value < 0.05 between HVA and EVA

**Fig. 1:**
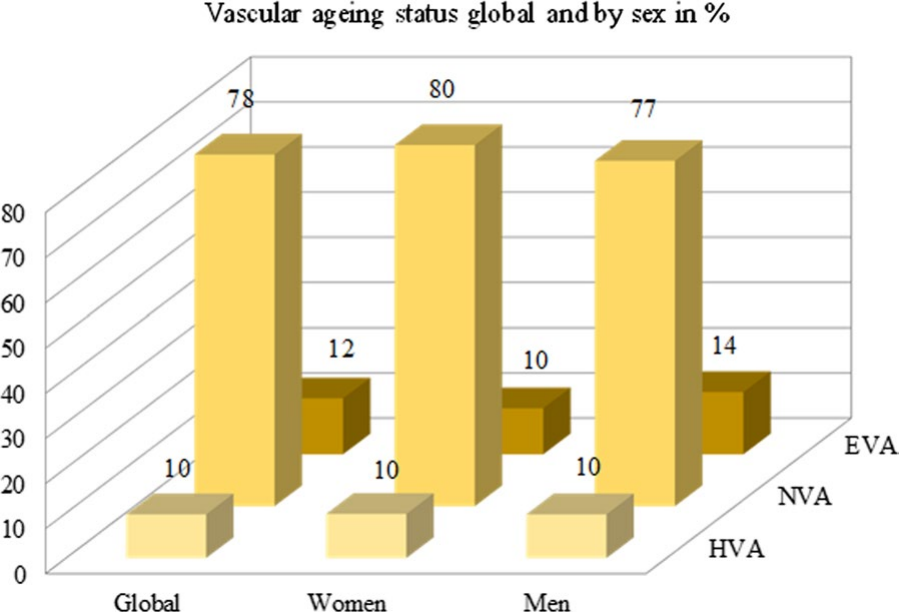
Vascular aging status global and by sex in percentage. *EVA* early vascular aging, *NVA* normal vascular aging, *HVA* healthy vascular aging

**Fig. 2:**
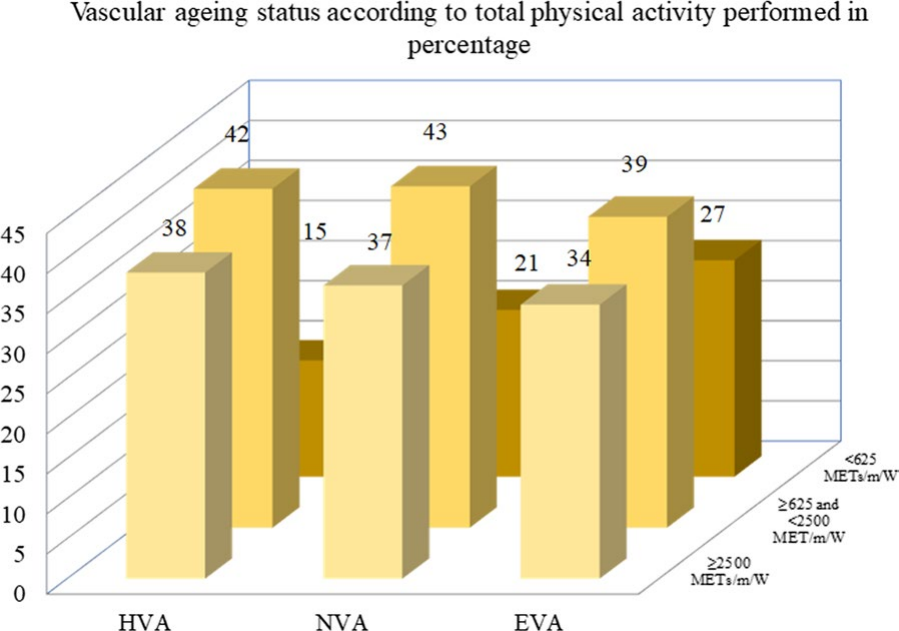
Vascular aging status according to total physical activity performed in percentage. METs/m/W, metabolic equivalents/min/week

The prevalence of individuals classified as HVA, NVA and EVA globally and by sex is shown in Fig. 1, finding differences between sex in the proportion of subjects in each category (*p* = 0.031). Subjects performing ≥ 2500 METs/m/W made up 36.4% of the sample, with 42.1% ≥ 625 and < 2500 METs/m/W and 21.5% < 625 METs/m/W. The distribution according to the degree of vascular aging is shown in Fig. 2, with no differences found between groups (*p* = 0.146).

### Association of arterial stiffness and vascular aging with physical activity and its intensity

In the multiple regression analysis, after controlling for possible confounding factors, the total physical activity showed a negative association with baPWV (*β* = − 0.045; 95% CI − 0.080 to − 0.009). Intense physical activity showed a negative relationship with baPWV (*β* = − 0.084; 95% CI − 0.136 to − 0.032), as can be observed in Table 3.

**Table 3:** Multiple regression analysis of arterial stiffness with physical activity

	β	95% CI	*p* value
Total			
M1			
Total PA, (METs/m/W)	− 0.045	− 0.080 to − 0.009	0.013
M2			
High-intensity PA, (METs/m/W)	− 0.084	− 0.136 to − 0.032	0.002
Moderate-intensity PA, (METs/m/W)	− 0.030	− 0.087 to 0.028	0.312
Low-intensity PA, (METs/m/W)	0.042	− 0.043 to 0.127	0.334
Men			
M1			
Total PA, (METs/m/W)	− 0.049	− 0.089 to − 0.009	0.017
M2			
High-intensity PA, (METs/m/W)	− 0.097	− 0.156 to − 0.039	0.001
Moderate-intensity PA, (METs/m/W)	− 0.024	− 0.089 to 0.040	0.463
Low-intensity PA, (METs/m/W)	0.059	− 0.049 to 0.166	0.283
Women			
M1			
Total PA, (METs/m/W)	− 0.021	− 0.099 to 0.057	0.595
M2			
High-intensity PA, (METs/m/W)	− 0.014	− 0.134 to 0.106	0.817
Moderate-intensity PA, (METs/m/W)	− 0.045	− 0.182 to 0.092	0.519
Low-intensity PA, (METs/m/W)	− 0.003	− 0.145 to 0.138	0.961

Model 1. Multiple regression analysis using baPWV m/s as dependent variables, total physical activity independent variable, and age (years), sex (woman = 1 and man = 2), years of smoker, alcohol consumption in g/week, Mediterranean diet adherence score, systolic blood pressure in mmHg; glycosylated hemoglobin in %, body mass index, antihypertensive drugs, lipid-lowering drugs and hypoglycaemic drugs (Yes = 1 and Non = 0) as adjustment variables

Model 2. Multiple regression analysis using baPWV m/s as dependent variables, high-intensity physical activity, moderate-intensity physical activity and low-intensity physical activity independent variable, and age (years), sex (woman = 1 and man = 2), years of smoker in years, alcohol consumption in g/week, Mediterranean diet adherence score, systolic blood pressure in mmHg; glycosylated hemoglobin in %, body mass index, antihypertensive drugs, lipid-lowering drugs and hypoglycaemic drugs (Yes = 1 and Non = 0) as adjustment variables

M1, model 1; M2, model 2; PA, physical activity; METs/m/W, metabolic equivalents/min/week

The results of the multinomial logistic regression analysis are presented in Table 4 and in Additional file 1: Fig. S1, globally and by sex. The OR of the total physical activity and the intense physical activity carried out by the subjects classified as NVA with respect to those classified as HVA was OR = 0.946; (95% CI 0.898 to 0.997) and OR = 0.903; (95% CI 0.840 to 0.971) and of those classified as EVA was OR = 0.916; (95% CI 0.852 to 0.986) and OR = 0.905; (95% CI 0.818 to 1.000). No association was found with moderate- or low-intensity physical activity. In the analysis by sex, there is no interaction of sex and physical activity with arterial stiffness or vascular aging.

**Table 4:** Association of physical activity with vascular aging

	Physical activity	OR	95% CI	*p* value
HVA (reference)				
Total				
M1	Total PA, (METs/m/W)	0.946	0.898 to 0.997	0.037
M2	High-intensity PA, (METs/m/W)	0.903	0.840 to 0.971	0.006
NVA	Moderate-intensity PA, (METs/m/W)	0.971	0.887 to 1.063	0.526
	Low-intensity PA, (METs/m/W)	1.079	0.928 to 1.255	0.325
M1	Total PA, (METs/m/W)	0.916	0.852 to 0.986	0.019
M2	High-intensity PA, (METs/m/W)	0.905	0.818 to 1.000	0.050
EVA	Moderate-intensity PA, (METs/m/W)	0.900	0.792 to 1.024	0.109
	Low-intensity PA, (METs/m/W)	1.035	0.852 to 1.256	0.731
Men				
M1	Total PA, (METs/m/W)	0.932	0.882 to 0.986	0.013
M2	High-intensity PA, (METs/m/W)	0.898	0.832 to 0.969	0.006
NVA	Moderate-intensity PA, (METs/m/W)	0.958	0.870 to 1.054	0.377
	Low-intensity PA, (METs/m/W)	1.033	0.866 to 1.230	0.716
M1	Total PA, (METs/m/W)	0.908	0.840 to 0.982	0.016
M2	High-intensity PA, (METs/m/W)	0.896	0.804 to 0.998	0.046
EVA	Moderate-intensity PA, (METs/m/W)	0.897	0.781 to 1.029	0.835
	Low-intensity PA, (METs/m/W)	1.020	0.814 to 1.277	0.121
Women				
M1	Total PA, (METs/m/W)	1.045	0.902 to 1.211	0.558
M2	High-intensity PA, (METs/m/W)	0.965	0.781 to 1.191	0.739
NVA	Moderate-intensity PA, (METs/m/W)	1.078	0.827 to 1.406	0.578
	Low-intensity PA, (METs/m/W)	1.194	0.892 to 1.597	0.233
M1	Total PA, (METs/m/W)	1.023	0.845 to 1.238	0.816
M2	High-intensity PA, (METs/m/W)	0.996	0.750 to 1.322	0.976
EVA	Moderate-intensity PA, (METs/m/W)	1.031	0.733 to 1.450	0.862
	Low-intensity PA, (METs/m/W)	1.102	0.757 to 1.603	0.614

Multinomial logistic regression analysis

Model 1. Multiple logistic regression analysis using vascular aging (HVA = 1; NVA = 2 and EVA = 3) as dependent variables (HVA reference), total physical as independent variable, as adjustment variables in the global analysis: age in years, consumption of alcohol in grams/week, years of smoking, sex (woman = 1 and man = 2), adherence to the Mediterranean diet, type 2 diabetes mellitus, arterial hypertension, obesity, antihypertensive drugs, lipid-lowering drugs and hypoglycaemic drugs (Yes = 1 and Non = 0) as adjustment variables). Model Fitting Criteria-2 Log Likelihood of Reduced Model

Model 2. Multiple logistic regression analysis using vascular aging (HVA = 1; NVA = 2 and EVA = 3) as dependent variables (HVA reference), high-intensity physical activity, moderate-intensity physical activity and low-intensity physical activity independent variable, as adjustment variables age in years, consumption of alcohol in grams/week,, years of smoking, sex (woman = 1 and man = 2), adherence to the Mediterranean diet, type 2 diabetes mellitus, arterial hypertension, obesity, antihypertensive drugs, lipid-lowering drugs and hypoglycaemic drugs (Yes = 1 and Non = 0) as adjustment variables. Model Fitting Criteria -2 Log Likelihood of Reduced Model

M1, Model 1: M2, Model 2; HVA, healthy vascular aging; NVA, normal vascular aging; EVA, early vascular aging; PA, physical activity; METs/m/W, metabolic equivalents/min/week

## Discussion

This study analyzed the association of physical activity and its intensity (measured with the short version of MLTPAQ) [38] with arterial stiffness and vascular aging in a Spanish population with intermediate cardiovascular risk. The results show that total physical activity and high intensity were negatively associated with arterial stiffness and improved the degree of vascular aging in the total sample.

Along these lines, previous studies have shown that regular physical activity and physical training delay the increase of arterial stiffness related to aging. Moreover, it has been observed that physical activity improves many other aspects of human health in different age groups, in healthy individuals and in those with cardiovascular risk factors, measured subjectively (through questionnaires) or objectively (using accelerometers), suggesting that arterial stiffness is negatively associated with regular physical activity [10–12, 26]. In the present study, this association was only found in men and with high-intensity physical activity, but results vary across different studies. Thus, Kozakova et al. [45] detected a reverse relationship between regular physical activity measured with an accelerometer and arterial stiffness in healthy adults in both men and women [45], although other authors have not found this association in women [46]. Similarly, and in agreement with the results of this study, the benefit of physical activity on arterial stiffness was only achieved when its intensity was high [14, 15, 47, 48]. Similarly, the recent study by Vandercappellen et al. [13] reported that practicing intense physical activity was associated with lower arterial stiffness, detecting no differences among different exercise patterns; thus, they concluded that, from the perspective of arterial stiffness, performing intense physical activity, regardless of the weekly pattern conducted, can be an important strategy to reduce the risk of cardiovascular disease. Moreover, the effects on arterial stiffness vary depending on the modality of physical activity performed. It has been reported that aerobic exercise has a beneficial effect on arterial stiffness, whereas strength exercise has different effects on arterial stiffness depending on the type and intensity of the exercise [49]. Likewise, a meta-analysis that reviewed 38 clinical trials, with a total of 2089 patients, concluded that aerobic and strength exercise reduced arterial stiffness [50]. In agreement with these results, previous studies have reported that greater physical activity (measured with an accelerometer) is associated with lower vascular stiffness [51]. Therefore, the current results suggest that regular physical activity is an effective index of arterial stiffness, and the effect of physical activity can be improved by increasing its intensity [10].

Similarly, in the present study, the beneficial effects of physical activity on arterial stiffness were confirmed on vascular aging. It is known that the regular and moderate-intense practice of physical activity in the general population improves vascular aging, as it reduces oxidative stress and preserves the bioavailability of NO [50, 52, 53]. In line with the results of this study, several authors have reported that healthy vascular aging is associated with greater physical activity [24, 53, 54]. Furthermore, regular aerobic exercise also improves the endothelial function with age in men, although this is not constant in post-menopausal women with estrogen deficiency. According to these findings, a certain, currently unknown estrogen blood level (greater than that found in post-menopausal women who are not being treated with estrogens) may be required for the physiological stimulus created by regular aerobic exercise to improve the endothelial function in post-menopausal women [32, 55, 56]. Furthermore, in women, reductions in circulating estrogens associated with menopause interact with other cellular aging processes to influence vascular function through mechanisms that, although not fully understood, also appear to involve oxidative stress and low-grade inflammation [32, 57–59]. However, the association of vascular aging with physical activity was not found in the Framingham study [28]. Therefore, considering the numerous benefits induced by regular physical activity, it seems logical that the harmful effects of aging on vascular function can be counteracted with the regular practice of physical activity [10].

The discrepancies found among the studies that analyzed the effect of physical activity and its intensity on arterial stiffness and aging may be caused by different factors: (1) samples of individuals of different ages, (2) physical activity measured with different methods (subjectively through questionnaires or objectively with accelerometers), and (3) arterial stiffness measured with different methods and using different devices.

This study has some limitations that must be highlighted. Firstly, the cross-sectional analysis does not allow causality to be inferred. Secondly, the results of this study only refer to the Spanish population of intermediate vascular risk, and they cannot be generalized to other groups or ethnicities. Thirdly, the physical activity was self-reported by the participants through validated questionnaires, that is, subjectively, since people tend to remember moderate and intense physical activity more accurately than low physical activity.

## Perspectives and significance

In clinical practice, health professionals may ask about the level and intensity of patients' physical activity performed in order to obtain more information that can help them better understand vascular health. In this study, the importance in vascular aging of the intensity of the physical activity performed is also described. For this purpose, more large-scale prospective cohort studies are needed to explore the process of vascular aging according to the amount and intensity of physical activity performed in adult patients and to analyze whether there are differences between the sexes, as well as the possible mechanisms involved in them.

## Conclusions

The results of this study suggest that, when intense physical activity is performed, the probability of presenting vascular aging is lower. In the analysis by sex, this association is only observed in men.

## Supplementary Information

**Additional file 1: Figure S1. ** Association of physical activity with vascular aging. Multinomial logistic regression analysis. (a), total. (b), men and (c), women. HPA: Physical Activity High intensity; MPA: Physical Activity Moderate intensity; LPA: Physical Activity Low intensity. HVA, healthy vascular aging; NVA, normal vascular aging; EVA, early vascular aging.

**Additional file 2: Table S1.** Percentile values of the baPWV by age and sex.

## Data Availability

The datasets used and/or analyzed during the current study are available from the corresponding author on reasonable request.

## Abbreviations

baPWV: Brachial–ankle pulse wave velocity
EVA: Early vascular aging
NVA: Normal vascular aging
HVA: Healthy vascular aging
MLTPAQ: Minnesota Leisure Time Physical Activity Questionnaire (MLTPAQ)
MET: Baseline metabolic equivalents
MD: Mediterranean diet
HPA: Physical Activity High intensity
MPA: Physical Activity Moderate intensity
LPA: Physical Activity Low intensity

## References

[1] Farris SG, Abrantes AM (2020). Mental health benefits from lifestyle physical activity interventions: a systematic review. Bull Menninger Clin.

[2] Manferdelli G, La Torre A, Codella R (2019). Outdoor physical activity bears multiple benefits to health and society. J Sports Med Phys Fitness.

[3] Malm C, Jakobsson J, Isaksson A (2019). Physical activity and sports-real health benefits: a review with insight into the public health of Sweden. Sports (Basel).

[4] Pedersen BK, Saltin B (2015). Exercise as medicine—evidence for prescribing exercise as therapy in 26 different chronic diseases. Scand J Med Sci Sports.

[5] Warburton DER, Bredin SSD (2017). Health benefits of physical activity: a systematic review of current systematic reviews. Curr Opin Cardiol.

[6] Guo Y, Shi H, Yu D, Qiu P (2016). Health benefits of traditional Chinese sports and physical activity for older adults: a systematic review of evidence. J Sport Health Sci.

[7] Nocon M, Hiemann T, Müller-Riemenschneider F, Thalau F, Roll S, Willich SN (2008). Association of physical activity with all-cause and cardiovascular mortality: a systematic review and meta-analysis. Eur J Cardiovasc Prev Rehabil.

[8] Füzéki E, Engeroff T, Banzer W (2017). Health benefits of light-intensity physical activity: a systematic review of accelerometer data of the National Health and Nutrition Examination Survey (NHANES). Sports Med.

[9] Ohkuma T, Ninomiya T, Tomiyama H, Kario K, Hoshide S, Kita Y, Inoguchi T, Maeda Y, Kohara K, Tabara Y (2017). Brachial-ankle pulse wave velocity and the risk prediction of cardiovascular disease: an individual participant data meta-analysis. Hypertension.

[10] Park W, Park HY, Lim K, Park J (2017). The role of habitual physical activity on arterial stiffness in elderly individuals: a systematic review and meta-analysis. J Exerc Nutr Biochem.

[11] Islam SJ, Beydoun N, Mehta A, Kim JH, Ko YA, Jin Q, Baltrus P, Topel ML, Liu C, Mujahid MS (2022). Association of physical activity with arterial stiffness among Black adults. Vasc Med.

[12] Tanaka H, Palta P, Folsom AR, Meyer ML, Matsushita K, Evenson KR, Aguilar D, Heiss G (2018). Habitual physical activity and central artery stiffening in older adults: the Atherosclerosis Risk in Communities study. J Hypertens.

[13] Vandercappellen EJ, Henry RMA, Savelberg H, van der Berg JD, Reesink KD, Schaper NC, Eussen S, van Dongen M, Dagnelie PC, Schram MT (2020). Association of the amount and pattern of physical activity with arterial stiffness: the Maastricht Study. J Am Heart Assoc.

[14] Königstein K, Infanger D, Klenk C, Carrard J, Hinrichs T, Schmidt-Trucksäss A (2020). Physical activity is favorably associated with arterial stiffness in patients with obesity and elevated metabolic risk. Int J Clin Pract.

[15] Figueroa A, Okamoto T, Jaime SJ, Fahs CA (2019). Impact of high- and low-intensity resistance training on arterial stiffness and blood pressure in adults across the lifespan: a review. Pflugers Arch.

[16] Collier SR, Frechette V, Sandberg K, Schafer P, Ji H, Smulyan H, Fernhall B (2011). Sex differences in resting hemodynamics and arterial stiffness following 4 weeks of resistance versus aerobic exercise training in individuals with pre-hypertension to stage 1 hypertension. Biol Sex Differ.

[17] Laurent S, Boutouyrie P, Cunha PG, Lacolley P, Nilsson PM (2019). Concept of extremes in vascular aging. Hypertension.

[18] Nowak KL, Rossman MJ, Chonchol M, Seals DR (2018). Strategies for achieving healthy vascular aging. Hypertension.

[19] Williams B, Mancia G, Spiering W, Agabiti Rosei E, Azizi M, Burnier M, Clement DL, Coca A, de Simone G, Dominiczak A (2018). 2018 ESC/ESH Guidelines for the management of arterial hypertension: The Task Force for the management of arterial hypertension of the European Society of Cardiology and the European Society of Hypertension: The Task Force for the management of arterial hypertension of the European Society of Cardiology and the European Society of Hypertension. J Hypertens.

[20] Gómez-Sánchez M, Gómez-Sánchez L, Patino-Alonso MC, Alonso-Domínguez R, Sánchez-Aguadero N, Recio-Rodríguez JI, González-Sánchez J, García-Ortiz L, Gómez-Marcos MA (2021). Relationship of healthy vascular aging with lifestyle and metabolic syndrome in the general Spanish population. The EVA study. Rev Esp Cardiol (Engl Ed).

[21] Groenewegen KA, den Ruijter HM, Pasterkamp G, Polak JF, Bots ML, Peters SA (2016). Vascular age to determine cardiovascular disease risk: a systematic review of its concepts, definitions, and clinical applications. Eur J Prev Cardiol.

[22] Neufingerl N, Cobain MR, Newson RS (2014). Web-based self-assessment health tools: who are the users and what is the impact of missing input information?. J Med Internet Res.

[23] Yang Q, Zhong Y, Ritchey M, Cobain M, Gillespie C, Merritt R, Hong Y, George MG, Bowman BA (2015). Vital signs: predicted heart age and racial disparities in heart age among U.S. adults at the state level. MMWR Morb Mortal Wkly Rep.

[24] Appiah D, Capistrant BD (2017). Cardiovascular disease risk assessment in the United States and low- and middle-income countries using predicted heart/vascular age. Sci Rep.

[25] Tabaei BP, Chamany S, Perlman S, Thorpe L, Bartley K, Wu WY (2019). Heart age, cardiovascular disease risk, and disparities by sex and race/ethnicity among New York City adults. Public Health Rep.

[26] Gomez-Sanchez M, Gomez-Sanchez L, Patino-Alonso MC, Cunha PG, Recio-Rodriguez JI, Alonso-Dominguez R, Sanchez-Aguadero N, Rodriguez-Sanchez E, Maderuelo-Fernandez JA, Garcia-Ortiz L (2020). Vascular aging and its relationship with lifestyles and other risk factors in the general Spanish population: early vascular ageing study. J Hypertens.

[27] Cunha PG, Cotter J, Oliveira P, Vila I, Boutouyrie P, Laurent S, Nilsson PM, Scuteri A, Sousa N (2015). Pulse wave velocity distribution in a cohort study: from arterial stiffness to early vascular aging. J Hypertens.

[28] Niiranen TJ, Lyass A, Larson MG, Hamburg NM, Benjamin EJ, Mitchell GF, Vasan RS (2017). Prevalence, correlates, and prognosis of healthy vascular aging in a western community-dwelling cohort: the Framingham heart study. Hypertension.

[29] Botto F, Obregon S, Rubinstein F, Scuteri A, Nilsson PM, Kotliar C (2018). Frequency of early vascular aging and associated risk factors among an adult population in Latin America: the OPTIMO study. J Hum Hypertens.

[30] Ji H, Teliewubai J, Lu Y, Xiong J, Yu S, Chi C, Li J, Blacher J, Zhang Y, Xu Y (2018). Vascular aging and preclinical target organ damage in community-dwelling elderly: the Northern Shanghai Study. J Hypertens.

[31] Nilsson PM, Laurent S, Cunha PG, Olsen MH, Rietzschel E, Franco OH, Ryliškytė L, Strazhesko I, Vlachopoulos C, Chen CH (2018). Characteristics of healthy vascular ageing in pooled population-based cohort studies: the global Metabolic syndrome and Artery REsearch Consortium. J Hypertens.

[32] Seals DR, Nagy EE, Moreau KL (2019). Aerobic exercise training and vascular function with ageing in healthy men and women. J Physiol.

[33] Marti R, Parramon D, Garcia-Ortiz L, Rigo F, Gomez-Marcos MA, Sempere I, Garcia-Regalado N, Recio-Rodriguez JI, Agudo-Conde C, Feuerbach N (2011). Improving interMediAte risk management. MARK study. BMC Cardiovasc Disord.

[34] Marrugat J, D'Agostino R, Sullivan L, Elosua R, Wilson P, Ordovas J, Solanas P, Cordón F, Ramos R, Sala J (2003). An adaptation of the Framingham coronary heart disease risk function to European Mediterranean areas. J Epidemiol Community Health.

[35] Conroy RM, Pyörälä K, Fitzgerald AP, Sans S, Menotti A, De Backer G, De Bacquer D, Ducimetière P, Jousilahti P, Keil U (2003). Estimation of ten-year risk of fatal cardiovascular disease in Europe: the SCORE project. Eur Heart J.

[36] Mancia G, Fagard R, Narkiewicz K, Redón J, Zanchetti A, Böhm M, Christiaens T, Cifkova R, De Backer G, Dominiczak A (2013). 2013 ESH/ESC guidelines for the management of arterial hypertension: the Task Force for the management of arterial hypertension of the European Society of Hypertension (ESH) and of the European Society of Cardiology (ESC). J Hypertens.

[37] World Medical Association (2013). World Medical Association Declaration of Helsinki: ethical principles for medical research involving human subjects. JAMA.

[38] Ruiz Comellas A, Pera G, Baena Díez JM, Mundet Tudurí X, Alzamora Sas T, Elosua R, Torán Monserrat P, Heras A, Forés Raurell R, Fusté Gamisans M (2012). Validation of a Spanish Short Version of the Minnesota Leisure Time Physical Activity Questionnaire (VREM). Rev Esp Salud Publica.

[39] Taylor HL, Jacobs DR, Schucker B, Knudsen J, Leon AS, Debacker G (1978). A questionnaire for the assessment of leisure time physical activities. J Chronic Dis.

[40] Elosua R, Garcia M, Aguilar A, Molina L, Covas MI, Marrugat J (2000). Validation of the minnesota leisure time physical activity questionnaire in Spanish women. Investigators of the MARATDON Group. Med Sci Sports Exerc.

[41] Elosua R, Marrugat J, Molina L, Pons S, Pujol E (1994). Validation of the minnesota leisure time physical activity questionnaire in Spanish men. The MARATHOM Investigators. Am J Epidemiol.

[42] Ainsworth BE, Haskell WL, Herrmann SD, Meckes N, Bassett DR, Tudor-Locke C, Greer JL, Vezina J, Whitt-Glover MC, Leon AS (2011). 2011 Compendium of Physical Activities: a second update of codes and MET values. Med Sci Sports Exerc.

[43] Nelson ME, Rejeski WJ, Blair SN, Duncan PW, Judge JO, King AC, Macera CA, Castaneda-Sceppa C (2007). Physical activity and public health in older adults: recommendation from the American College of Sports Medicine and the American Heart Association. Med Sci Sports Exerc.

[44] Shirai K, Hiruta N, Song M, Kurosu T, Suzuki J, Tomaru T, Miyashita Y, Saiki A, Takahashi M, Suzuki K (2011). Cardio-ankle vascular index (CAVI) as a novel indicator of arterial stiffness: theory, evidence and perspectives. J Atheroscler Thromb.

[45] Kozakova M, Palombo C, Mhamdi L, Konrad T, Nilsson P, Staehr PB, Paterni M, Balkau B (2007). Habitual physical activity and vascular aging in a young to middle-age population at low cardiovascular risk. Stroke.

[46] Tanaka H, DeSouza CA, Seals DR (1998). Absence of age-related increase in central arterial stiffness in physically active women. Arterioscler Thromb Vasc Biol.

[47] Sugawara J, Otsuki T, Tanabe T, Hayashi K, Maeda S, Matsuda M (2006). Physical activity duration, intensity, and arterial stiffening in postmenopausal women. Am J Hypertens.

[48] Gando Y, Yamamoto K, Murakami H, Ohmori Y, Kawakami R, Sanada K, Higuchi M, Tabata I, Miyachi M (2010). Longer time spent in light physical activity is associated with reduced arterial stiffness in older adults. Hypertension.

[49] Li Y, Hanssen H, Cordes M, Rossmeissl A, Endes S, Schmidt-Trucksäss A (2015). Aerobic, resistance and combined exercise training on arterial stiffness in normotensive and hypertensive adults: a review. Eur J Sport Sci.

[50] Zhang Y, Qi L, Xu L, Sun X, Liu W, Zhou S, van de Vosse F, Greenwald SE (2018). Effects of exercise modalities on central hemodynamics, arterial stiffness and cardiac function in cardiovascular disease: systematic review and meta-analysis of randomized controlled trials. PLoS ONE.

[51] Andersson C, Lyass A, Larson MG, Spartano NL, Vita JA, Benjamin EJ, Murabito JM, Esliger DW, Blease SJ, Hamburg NM (2015). Physical activity measured by accelerometry and its associations with cardiac structure and vascular function in young and middle-aged adults. J Am Heart Assoc.

[52] Antunes BM, Rossi FE, Cholewa JM, Lira FS (2016). Regular physical activity and vascular aging. Curr Pharm Des.

[53] Kucharska-Newton AM, Stoner L, Meyer ML (2019). Determinants of vascular age: an epidemiological perspective. Clin Chem.

[54] Laurent S, Boutouyrie P, Cunha PG, Lacolley P, Nilsson PM (2019). Concept of extremes in vascular aging. Hypertension.

[55] Faulkner JL, Belin de Chantemèle EJ (2019). Sex hormones, aging and cardiometabolic syndrome. Biol Sex Differ.

[56] Moreau KL, Babcock MC, Hildreth KL (2020). Sex differences in vascular aging in response to testosterone. Biol Sex Differ.

[57] Moreau KL, Stauffer BL, Kohrt WM, Seals DR (2013). Essential role of estrogen for improvements in vascular endothelial function with endurance exercise in postmenopausal women. J Clin Endocrinol Metab.

[58] Moreau KL, Hildreth KL (2014). Vascular aging across the menopause transition in healthy women. Adv Vasc Med.

[59] Hildreth KL, Kohrt WM, Moreau KL (2014). Oxidative stress contributes to large elastic arterial stiffening across the stages of the menopausal transition. Menopause.

