# Supplementary material for: Association of physical activity with vascular aging in a population with intermediate cardiovascular risk, analysis by sex: MARK study
Source: Biol Sex Differ. 2022 Aug 20;13:46. doi: 10.1186/s13293-022-00456-w (PMC9392339; doi:10.1186/s13293-022-00456-w)
Supplement: Supplementary file 1 [file 13293_2022_456_MOESM2_ESM.odt]

Table S1. Percentile values of the baPWV by age and sex.

| Age in year | 30-40 | 41-50 | 51-60 | 61-70 | 71-80 |
| --- | --- | --- | --- | --- | --- |
| Men 90 th | 14.77 | 15.36 | 16.84 | 18.71 | 20.24 |
| Men 10 th | 9.57 | 10.81 | 11.69 | 12.55 | 13.88 |
| Women 90 th | 14.47 | 15.36 | 17.04 | 18.45 | 19.79 |
| Women 10 th | 10.12 | 11.28 | 11.76 | 12.63 | 13.59 |

baPWV, brachial-ankle pulse wave velocity
